# Hypoxic-Ischemic Encephalopathy in Newborns: Pathophysiology, Early Identification, and Management

**DOI:** 10.7759/cureus.104079

**Published:** 2026-02-22

**Authors:** Maryam S Alhosani, Giorgi Sakvarelidze

**Affiliations:** 1 Department of Medicine, University of Georgia, Tbilisi, GEO; 2 Department of Neurology, University of Georgia, Tbilisi, GEO

**Keywords:** hypoxic-ischemic encephalopathy, neonatal birth asphyxia, neonatal brain injury, neonatal hypoxic-ischemic encephalopathy, newborn, perinatal hypoxia-ischemia, primary energy failure, sarnat staging of neonatal encephalopathy, secondary energy failure, therapeutic hypothermia

## Abstract

Hypoxic-ischemic encephalopathy (HIE) is a severe neurological condition resulting from impaired oxygen delivery and reduced cerebral blood flow to the neonatal brain during the perinatal period. It remains one of the leading causes of neonatal mortality and long-term neurodevelopmental disability worldwide, despite significant advances in obstetric and neonatal care. The pathophysiology of HIE is complex and evolves through a cascade of events beginning with primary energy failure, followed by secondary energy failure characterized by mitochondrial dysfunction, oxidative stress, inflammation, excitotoxicity, and delayed neuronal cell death. Early identification of affected neonates is critical, as timely intervention can significantly influence neurological outcomes. Diagnosis relies on a combination of clinical assessment, neuroimaging, particularly magnetic resonance imaging (MRI), and neurophysiological monitoring such as amplitude-integrated electroencephalography (aEEG), which aid in determining severity and prognosis. Therapeutic hypothermia has emerged as the cornerstone of neuroprotective treatment and has demonstrated improved survival and neurodevelopmental outcomes when initiated within the first six hours of life. Additional supportive strategies, including seizure control and meticulous metabolic and hemodynamic management, play an essential role in comprehensive care. This review summarizes the current understanding of the pathophysiology, diagnostic approaches, and management strategies of HIE in newborns, emphasizing the importance of early intervention and ongoing research into novel neuroprotective therapies aimed at improving long-term outcomes.

## Introduction and background

Hypoxic-ischemic encephalopathy (HIE) is a devastating form of neonatal brain injury that results from reduced cerebral blood flow and insufficient oxygen delivery to the brain, most commonly occurring during the perinatal period. It represents one of the most serious complications of neonatal asphyxia and remains a major contributor to neonatal mortality and long-term neurological morbidity worldwide. Survivors of HIE often experience persistent neurodevelopmental impairments, including cerebral palsy, epilepsy, cognitive deficits, and behavioral disorders, which significantly affect quality of life [1].

The pathogenesis of HIE is complex and involves a dynamic sequence of biochemical and molecular events rather than a single acute insult. The initial hypoxic-ischemic event leads to primary energy failure, characterized by impaired oxidative metabolism and depletion of high-energy phosphates. This is followed by a secondary phase of injury that develops hours to days later and is driven by reperfusion injury, excessive glutamate release, oxidative stress, mitochondrial dysfunction, inflammatory responses, and activation of programmed cell death pathways. These interconnected mechanisms ultimately result in progressive neuronal and glial injury [1].

Despite advances in perinatal and neonatal care, therapeutic hypothermia remains the only established standard treatment for neonatal HIE. While hypothermia has been shown to reduce mortality and improve neurodevelopmental outcomes, its neuroprotective effects are limited, and a significant proportion of treated infants continue to develop long-term neurological disabilities [1-3]. This has prompted increasing interest in adjunctive and emerging therapies, including pharmacological agents and regenerative approaches such as stem cell therapy, which aim to target multiple pathways involved in secondary brain injury.

A deeper understanding of the underlying pathophysiological mechanisms of HIE is essential for the development of more effective therapeutic strategies. Ongoing research continues to explore novel neuroprotective interventions that may enhance the efficacy of hypothermia and improve neurological outcomes for affected newborns. This growing body of evidence highlights the need for continued investigation into both the mechanisms and management of HIE.

## Review

Methods

A narrative literature review was conducted to summarize current evidence regarding the epidemiology, etiology, pathophysiology, diagnosis, and management of HIE in newborns. An electronic search of the PubMed, Scopus, and Google Scholar databases was performed to identify relevant studies published in English. The search strategy included combinations of the following keywords: "hypoxic-ischemic encephalopathy", "neonatal brain injury", "perinatal asphyxia", "therapeutic hypothermia", and "neuroprotection".

Articles published within the past 10-15 years were prioritized to ensure inclusion of contemporary evidence, although seminal earlier studies were also reviewed when relevant. Eligible publications included review articles, randomized controlled trials, observational studies, and clinical guidelines focusing on neonatal HIE. Studies were excluded if they were not relevant to neonatal populations, lacked clinical relevance, or were duplicate publications.

Titles and abstracts were initially screened for relevance, followed by a full-text review of selected articles. Additional references were identified through manual screening of the bibliographies of relevant articles. The included literature was qualitatively synthesized to provide a comprehensive overview of current understanding and management approaches for HIE in newborns.

Epidemiology and etiopathogenesis of HIE

HIE is a major cause of neonatal morbidity and mortality worldwide, with incidence varying significantly by region. In high-income countries, HIE occurs in approximately 1-2 per 1,000 live births, largely due to advances in prenatal care, intrapartum monitoring, and neonatal resuscitation. However, in low- and middle-income countries, the incidence is substantially higher, ranging from 10 to 20 per 1,000 live births, reflecting disparities in access to obstetric and neonatal care [4].

Despite improvements in perinatal management, HIE continues to be associated with significant long-term neurological sequelae, including cerebral palsy, epilepsy, and cognitive impairment [5]. Although therapeutic hypothermia has improved outcomes in selected cases, its neuroprotective effects remain limited, and a considerable proportion of affected infants develop persistent neurodevelopmental disabilities. These epidemiological trends highlight the global burden of HIE and emphasize the need for effective preventive and therapeutic strategies.

Neonatal HIE primarily results from perinatal asphyxia, a condition characterized by impaired gas exchange leading to insufficient oxygen delivery and reduced cerebral perfusion around the time of birth. The most commonly implicated etiological factors are acute obstetric complications that compromise fetal oxygenation. These include placental abruption, umbilical cord prolapse, and uterine rupture, all of which can abruptly interrupt placental blood flow and precipitate hypoxic-ischemic injury to the neonatal brain.

The severity of brain injury is influenced by both the duration and intensity of hypoxia-ischemia, as well as the newborn’s ability to initiate effective respiration at birth. In severe cases, prolonged oxygen deprivation leads to metabolic acidosis and widespread neuronal dysfunction, contributing to disturbances in consciousness, reflexes, and motor tone. Thus, HIE represents the neurological consequence of a critical failure in oxygen delivery during the perinatal period, most often secondary to identifiable intrapartum events [6,7].

Pathophysiology of HIE

**Figure 1:**
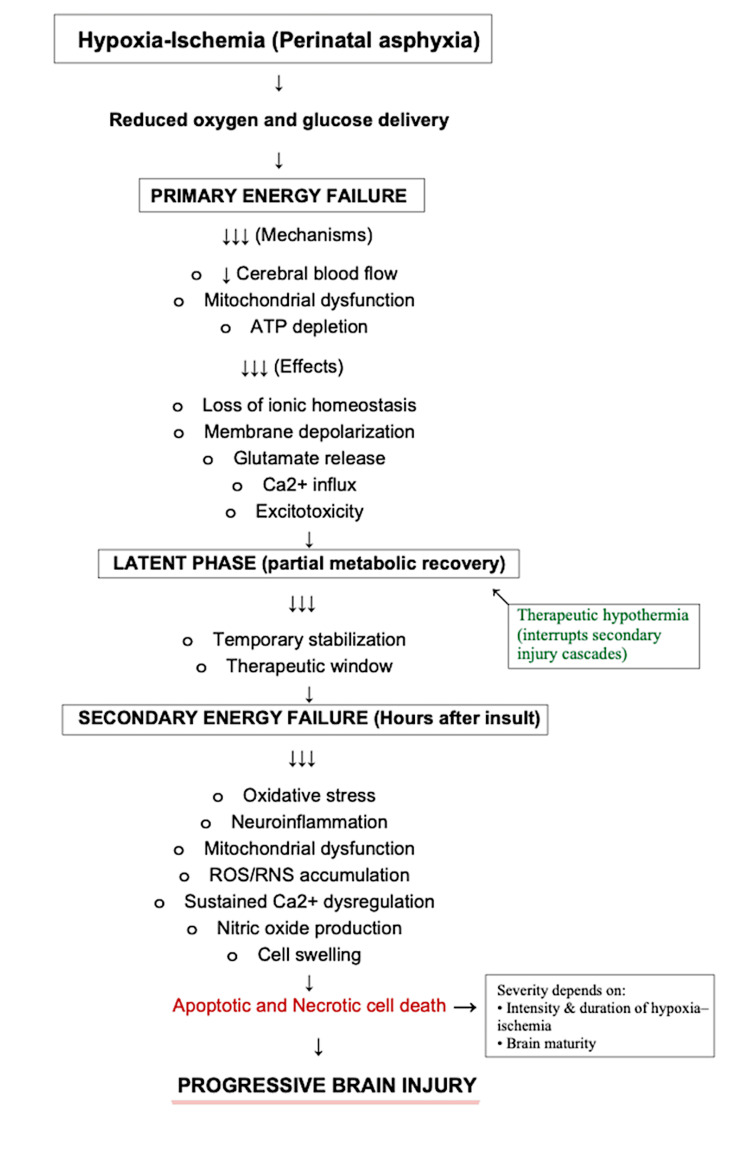
Pathophysiological cascade of HIE. Perinatal asphyxia leads to reduced oxygen and glucose delivery to the neonatal brain, resulting in a primary phase of energy failure characterized by impaired mitochondrial oxidative phosphorylation, adenosine triphosphate depletion, excitotoxic neurotransmitter release, and intracellular calcium influx. Following a brief latent phase of partial metabolic recovery, a delayed secondary energy failure develops, driven by progressive mitochondrial dysfunction, oxidative stress, neuroinflammation, and sustained calcium dysregulation, ultimately culminating in apoptotic and necrotic neuronal cell death. Recognition of this latent phase provides the biological rationale for therapeutic hypothermia to interrupt secondary injury cascades and limit irreversible brain injury. In this schematic, single arrows indicate sequential progression between pathophysiological stages, whereas triple arrows denote a single upstream process leading to multiple downstream mechanisms or effects. Bullet points represent parallel processes occurring within the same pathophysiological phase. HIE: hypoxic-ischemic encephalopathy; ATP: adenosine triphosphate; Ca²⁺: calcium ion; ROS: reactive oxygen species; RNS: reactive nitrogen species. This schematic represents an author-generated synthesis of the pathophysiological cascade of HIE, constructed according to and informed by previously published literature [8,9].

HIE arises from a critical reduction in oxygen and glucose delivery to the neonatal brain, typically secondary to perinatal asphyxia. This deprivation disrupts oxidative metabolism, leading to a cascade of cellular and molecular events that culminate in neuronal injury and death. The pattern of brain injury in HIE is classically described as occurring in two sequential phases of energy failure (Figure 1).

The primary energy failure occurs during the hypoxic-ischemic insult itself. Reduced cerebral blood flow and oxygen availability impair mitochondrial oxidative phosphorylation, resulting in rapid depletion of adenosine triphosphate (ATP). This energy failure causes loss of ionic homeostasis, membrane depolarization, and excessive release of excitatory neurotransmitters, particularly glutamate. Overactivation of glutamate receptors leads to intracellular calcium influx, triggering enzymatic pathways that damage cellular membranes, proteins, and DNA.

Following a brief latent period during which partial metabolic recovery may occur, a secondary energy failure develops hours after the initial insult. This delayed phase is characterized by mitochondrial dysfunction, oxidative stress, and neuroinflammation. Accumulation of reactive oxygen and nitrogen species promotes lipid peroxidation, while sustained calcium dysregulation and nitric oxide production further exacerbate neuronal injury. These processes ultimately result in apoptotic and necrotic cell death, extending the extent of brain damage beyond the initial ischemic event.

The severity and distribution of injury depend on the intensity and duration of hypoxia-ischemia, as well as the maturity of the brain at the time of insult. Clinically, neurological manifestations evolve over the first 72 hours of life, reflecting the progression of these underlying pathophysiological mechanisms. The recognition of this delayed secondary phase provides the rationale for therapeutic hypothermia, which aims to interrupt these injurious cascades during the narrow window of opportunity following birth and thereby limit irreversible brain injury [8,9].

Clinical presentation and severity classification

The clinical presentation of neonatal HIE varies according to the severity and duration of the hypoxic-ischemic insult. Clinical manifestations may be evident immediately after birth or may evolve progressively during the first hours of life.

Mild to moderate manifestations of HIE in newborns commonly include abnormal muscle tone, presenting as hypotonia or hypertonia, feeding difficulties, irritability, fatigue, and a weak or diminished cry. Affected infants may also exhibit altered skin coloration, including pallor or a bluish or gray discoloration of the skin, lips, and extremities, indicative of compromised oxygenation. Neurological findings at this stage are often variable and may fluctuate during the early postnatal period.

Severe HIE is characterized by significant neurological and systemic dysfunction. Clinical features may include episodes of apnea, impaired or absent reflexes, irregular or slowed heart rate (bradycardia), and altered levels of consciousness ranging from stupor to coma. Seizures are frequently observed within the first 24 hours of life and represent a key indicator of ongoing cerebral injury [9]. In the most severe cases, infants may be unable to maintain adequate ventilation or cardiovascular stability without intensive supportive care. The pattern, combination, and progression of these clinical signs are essential for assessing disease severity and guiding further diagnostic evaluation and appropriate management strategies [9-11].

Sarnat staging of neonatal encephalopathy

The severity of HIE is commonly classified using the Sarnat and Sarnat staging system, which categorizes neonatal encephalopathy into three stages based on neurological examination and clinical findings (Table 1) [11]. This assessment involves evaluation of multiple neurological parameters, including mental status, reflexes, muscle tone, autonomic function (including pupillary response and other autonomic indicators), seizure activity, and electroencephalographic (EEG) patterns. This classification system plays a crucial role in guiding treatment decisions, predicting neurological outcomes, and determining eligibility for therapeutic hypothermia.

**Table 1:** Severity-related clinical features derived from prior descriptions of neonatal encephalopathy. The framework was restructured to enable early bedside evaluation after birth. Neurological findings were assessed across several functional domains, and the overall pattern of abnormalities was used to determine severity. Distinctions between adjacent severity categories may not always be absolute. The table was created by the authors and synthesized from established pathophysiological mechanisms described in the literature [11,12].

Domain	Stage I (Mild)	Stage II (Moderate)	Stage III (Severe)
Consciousness	Hyperalert	Lethargy	Coma/stupor
Muscle tone	Normal or mildly increased	Hypotonia or Hypertonia	Flaccid or rigid tone
Pupillary response	Mydriasis or dilated pupils with preserved reactivity	Myosis or constricted pupils	Poorly reactive or non-reactive pupils
Seizure activity	No seizures or typically absent	Common; often multifocal seizures	May be frequent or difficult to detect due to profound cerebral suppression
EEG patterns	Generally normal background activity	Abnormal background with periodic or slow-wave patterns	Severely suppressed activity or near-isoelectric tracing
Respiratory patterns	Rapid breathing or hyperventilation	Periodic or irregular respirations	Apnea or dependence on ventilator support

Stage I (Mild HIE)

Infants with mild HIE are typically hyperalert or irritable and may exhibit an increased startle response. Muscle tone is often normal or mildly increased, and primitive reflexes, such as the Moro and suck reflexes, remain intact. Seizures are uncommon at this stage. Autonomic function is characterized by sympathetic dominance, including findings such as dilated pupils (mydriasis). EEG findings are usually normal. Clinical symptoms generally resolve within 24 hours, and long-term neurological outcomes are generally favorable; however, some infants may still develop subtle neurodevelopmental impairments, particularly affecting cognitive function.

Stage II (Moderate HIE)

Moderate HIE is characterized by lethargy, hypotonia, diminished spontaneous activity, and weakened primitive reflexes. Seizures are common and frequently occur within the first 24 hours of life. Infants may also present with abnormal breathing patterns, feeding difficulties, and autonomic dysfunction characterized by parasympathetic dominance, such as pupillary constriction (miosis). EEG findings are abnormal and often demonstrate low-voltage activity or theta-delta patterns. The duration of this stage typically ranges from 2 to 14 days. Infants with moderate HIE require close neurological monitoring and therapeutic hypothermia when eligible; however, close monitoring is also recommended in mild HIE, as neurological status may evolve during the early postnatal period.

Stage III (Severe HIE)

Severe HIE is associated with profound neurological depression, including severely impaired consciousness, flaccid muscle tone, absent reflexes, and minimal spontaneous movement. Seizures may be frequent and refractory. EEG findings are markedly abnormal, often showing severely suppressed or isoelectric patterns. Infants with severe HIE commonly require intensive supportive care, and the prognosis is poor, with a high risk of mortality or severe long-term neurological disability [11,12].

Other associated complications of HIE

HIE is associated with a broad range of long-term neurological complications that may persist beyond the neonatal period. The extent of chronic neurological impairment is largely determined by the severity and duration of the initial hypoxic-ischemic insult. Survivors may experience outcomes ranging from subtle cognitive deficits to profound motor and cognitive disabilities that significantly limit independence. Long-term sequelae commonly reported include epilepsy, movement disorders, myoclonus, cognitive dysfunction, and disorders of consciousness. In severe cases, persistent vegetative states or severe global neurological impairment may occur. Early prediction of long-term neurological outcomes remains challenging, highlighting the need for prolonged follow-up and comprehensive neurodevelopmental assessment in affected infants [13].

Multiple maternal, intrapartum, and neonatal factors have been associated with the development and severity of HIE, as well as with subsequent neurological outcomes. Maternal factors such as advanced maternal age, underlying medical conditions, medication use during pregnancy, and adverse socioeconomic conditions have been implicated in increasing vulnerability to perinatal hypoxia. Intrapartum complications, including pregnancy-related hypertensive disorders and reduced amniotic fluid volume, further contribute to the risk of hypoxic-ischemic injury. Postnatally, the need for extensive resuscitation at birth and evidence of metabolic acidosis are frequently associated with more severe disease. Neuroimaging patterns, particularly involvement of deep gray matter structures or watershed regions, have also been linked to outcome variability. These findings underscore the complex interplay between antenatal, peripartum, and neonatal factors in influencing both the occurrence of HIE and its clinical trajectory, highlighting the importance of early identification of high-risk infants to guide timely intervention and long-term follow-up [14].

Diagnostic evaluation

The diagnosis of HIE is based on a combination of perinatal history, clinical findings, neurological examination, and laboratory and imaging studies. Early and accurate diagnosis is essential, as timely intervention, particularly therapeutic hypothermia, can significantly improve neurological outcomes [15,16].

A detailed perinatal history is the first step in evaluation and includes evidence of fetal distress, perinatal asphyxia, and low Apgar scores assessed at 5 and 10 minutes after birth. Apgar scoring evaluates appearance, pulse, grimace, activity, and respiration and provides an early indication of neonatal compromise. These findings support early risk stratification but are not diagnostic on their own.

Neurological examination remains central to the diagnostic process. Assessment includes level of consciousness, muscle tone, spontaneous activity, primitive reflexes, respiratory effort, and the presence of seizures. These clinical features assist in determining disease severity and help classify infants according to established staging systems such as the Sarnat classification.

Laboratory investigations support the diagnosis by identifying metabolic and systemic abnormalities associated with hypoxic injury. Arterial blood gas analysis may demonstrate severe metabolic acidosis, commonly defined by a pH less than 7.0 or a base deficit equal to or greater than 12 mmol/L. Elevated lactate levels and evidence of multiorgan dysfunction further reflect the severity of hypoxic-ischemic insult.

EEG and amplitude-integrated electroencephalography (aEEG) play a critical role in diagnostic evaluation by detecting both clinical and subclinical seizure activity and by assessing background cerebral function. Abnormal EEG or aEEG patterns, including low-voltage activity, discontinuous backgrounds, or burst suppression, are associated with more severe brain injury and poorer neurodevelopmental outcomes. Continuous neurophysiological monitoring is particularly valuable in infants undergoing therapeutic hypothermia.

Neuroimaging is an essential component of diagnostic assessment. Cranial ultrasound may be used as an initial bedside tool; however, magnetic resonance imaging (MRI) provides superior sensitivity for defining the extent and pattern of brain injury. Diffusion-weighted imaging is especially useful in identifying early ischemic changes and in determining injury severity. Specific patterns, such as involvement of deep gray matter structures or watershed regions, have been associated with outcome variability.

In addition to neurological findings, systemic involvement should be assessed as part of the diagnostic workup. Evidence of renal or metabolic dysfunction has been associated with more severe disease and poorer prognosis, highlighting the importance of comprehensive multi-system evaluation.

Overall, the integration of clinical assessment, laboratory findings, neurophysiological monitoring, and neuroimaging provides a comprehensive diagnostic approach that guides management decisions and assists in predicting long-term neurological outcomes in infants with HIE.

Management strategies

Management of HIE focuses on early stabilization, neuroprotection, seizure control, and prevention of secondary brain injury. Prompt identification of eligible infants and timely initiation of therapeutic hypothermia remain the cornerstone of treatment for moderate to severe HIE [17].

Initial management includes optimization of airway, breathing, and circulation to ensure adequate oxygenation and cerebral perfusion. Careful control of blood glucose, electrolytes, blood pressure, and acid-base balance is essential to minimize secondary metabolic injury. Seizures should be promptly identified and treated, as ongoing seizure activity is associated with worse neurological outcomes.

*Therapeutic hypothermia* is currently the only evidence-based neuroprotective therapy shown to improve outcomes in neonates with moderate to severe HIE. Standard protocols aim to achieve a target core temperature of approximately 33.5°C for a duration of 72 hours, followed by gradual rewarming. This intervention has been shown to reduce mortality and long-term neurodevelopmental disability when initiated within the first six hours of life.

Recent evidence suggests that impaired thermoregulation in neonates with HIE may provide additional prognostic information during therapeutic hypothermia. Infants with more severe hypoxic-ischemic injury often exhibit spontaneous hypothermia and therefore require less active cooling to maintain the target temperature. Studies have demonstrated that neonates with unfavorable short-term outcomes required lower cooling device output to sustain core temperatures between 33 and 34°C compared to those with more favorable outcomes. This reduced need for active cooling has been strongly associated with higher MRI injury scores and increased mortality.

Cooling device output temperature has therefore emerged as a potential early physiological biomarker of injury severity during therapeutic hypothermia. Greater impairment in temperature regulation reflects more extensive cerebral injury and may aid in early risk stratification. This association underscores the importance of continuous temperature monitoring and careful adjustment of cooling parameters throughout treatment.

Neuroimaging, particularly MRI with diffusion-weighted sequences performed during the first week of life, plays a crucial role in guiding prognosis and ongoing management. A higher injury burden on MRI correlates with poorer outcomes and may influence decisions regarding escalation of supportive care and long-term follow-up planning.

Management of HIE extends beyond the acute phase and requires a multidisciplinary approach. Infants with moderate to severe disease should undergo long-term neurodevelopmental surveillance to detect cognitive, motor, and behavioral impairments early. Ongoing research into adjunctive neuroprotective therapies aims to enhance the benefits of therapeutic hypothermia and improve outcomes across all severity grades, including mild HIE.

*Supportive care* in the neonatal intensive care unit (NICU) is a fundamental component of management for infants with HIE. Many affected neonates require respiratory support due to impaired respiratory drive, apnea, or inadequate gas exchange. Supplemental oxygen or mechanical ventilation may be necessary to maintain appropriate oxygenation and carbon dioxide levels, thereby preventing secondary hypoxic injury [18].

Hemodynamic stability is carefully maintained through continuous monitoring of heart rate and blood pressure, judicious fluid administration, and the use of inotropic agents when indicated. Maintaining adequate cerebral perfusion is essential to limit ongoing neuronal damage. Seizure management is a critical aspect of supportive care, as seizures are common in neonates with HIE and are associated with worse neurological outcomes. Anticonvulsant therapy is initiated promptly, with agents such as phenobarbital or levetiracetam commonly used to control both clinical and electrographic seizures.

Ongoing monitoring of vital signs, oxygen saturation, and core temperature is essential throughout treatment to ensure physiologic stability and to minimize secondary brain injury.

Moreover, broader healthcare system factors may influence the management and evaluation of HIE, as highlighted during the COVID-19 pandemic. Disruptions in prenatal and perinatal care, changes in obstetric practices, and increased maternal comorbidities were associated with a rise in NICU admissions for HIE evaluation during this period. Despite the increased number of infants screened for suspected HIE, the proportion of neonates ultimately diagnosed with HIE or treated with therapeutic hypothermia remained comparable to pre-pandemic levels. These observations emphasize the importance of maintaining standardized screening protocols, timely neurological assessment, and access to therapeutic hypothermia, even during periods of healthcare system strain. Ensuring continuity of neonatal neurocritical care pathways is essential to prevent delays in diagnosis and to preserve outcomes for infants at risk of hypoxic-ischemic brain injury [19].

Additionally, *nutritional support *plays an important role in the overall management of neonates with HIE [20,21]. In the acute phase, enteral feeding is often delayed due to hemodynamic instability or impaired gastrointestinal function. However, emerging evidence suggests that minimal enteral nutrition (trophic feeding) during therapeutic hypothermia is safe and beneficial in clinically stable neonates [22]. During this period, nutritional requirements are met through intravenous fluids to maintain hydration and provide essential glucose and electrolytes.

*Enteral feeding* via tube feeding is initiated once the infant is clinically stable and oral feeding is considered unsafe. The transition to oral feeding is guided by neurological status and swallowing function. Careful metabolic management is essential to reduce additional neurological stress. Hypoglycemia, electrolyte imbalances, and metabolic acidosis are promptly corrected, as these disturbances can exacerbate neuronal injury.

Continuous monitoring and maintenance of adequate oxygen saturation, hemodynamic stability, glycemic control, and normothermia are essential to minimizing secondary brain injury and promoting recovery during both the acute and subacute phases of care. In parallel, several adjunctive neuroprotective strategies are under investigation, including erythropoietin (EPO), xenon gas, and stem cell-based therapies. Although these approaches show promise, their use remains experimental. Early involvement of a multidisciplinary team, encompassing neonatologists, neurologists, and developmental specialists, is strongly recommended to optimize ongoing management and long-term follow-up [23-25].

As affected children grow, some complications may emerge, necessitating additional supportive interventions. These may include physical or occupational therapy, speech and language therapy, and the use of assistive devices such as corrective lenses or hearing aids, which can be implemented to support functional development. Overall, the integration of therapeutic hypothermia with comprehensive supportive care continues to represent the cornerstone of HIE management, with the overarching aim of improving long-term neurological outcomes and quality of life for affected infants.

Prognosis and long-term outcomes

Long-term outcomes following HIE remain a major source of morbidity despite advances in neonatal care. Survivors may experience persistent impairments across multiple neurodevelopmental domains, including motor function, cognition, behavior, and language. Cerebral palsy, epilepsy, learning difficulties, and executive dysfunction are among the most commonly reported sequelae, with risk increasing in parallel with the severity of the initial encephalopathic insult. Although therapeutic hypothermia has significantly improved survival and neurodevelopmental outcomes in term and late-preterm infants with moderate to severe HIE, a substantial proportion of affected infants continue to demonstrate long-term disabilities. Moreover, current evidence does not support a clear long-term benefit of adjunctive neuroprotective therapies when combined with hypothermia, and their role remains under investigation. Ongoing longitudinal follow-up and standardized neurodevelopmental assessment are therefore essential to accurately characterize outcomes, guide early intervention strategies, and optimize quality of life for children affected by HIE [26,27].

Furthermore, evidence from landmark randomized controlled trials, including the NICHD, TOBY, and CoolCap studies, has been central to defining long-term outcomes and establishing therapeutic hypothermia as the standard of care for term infants with HIE. These trials introduced strict inclusion criteria and standardized treatment protocols, representing a major methodological advancement compared with earlier pre-cooling era studies.

In the pivotal NICHD Neonatal Research Network trial, 102 infants were randomized to whole-body hypothermia and 106 to standard supportive care. The composite outcome of death or moderate to severe disability occurred in 44% of infants treated with hypothermia compared with 62% in the control group (p = 0.01). Mortality was lower in the hypothermia group (24%) than in controls (37%), although this difference did not reach statistical significance (p = 0.08). Neurodevelopmental assessment at 18-22 months demonstrated no significant differences between groups in rates of cerebral palsy, blindness, or hearing impairment requiring aids.

Long-term follow-up of the same cohort at 6-7 years of age confirmed sustained survival benefits associated with hypothermia. Death occurred in 28% of children in the hypothermia group compared with 44% in the control group (p = 0.04), while the combined outcome of death or severe disability was observed in 41% versus 60%, respectively (p = 0.03). Although rates of moderate to severe disability, attention-deficit/hyperactivity disorder, and visuospatial dysfunction among survivors were similar between groups, hypothermia significantly reduced mortality and the risk of the combined endpoint of death or an IQ score below 70 [28].

Emerging evidence suggests that biological sex may significantly influence injury mechanisms and recovery trajectories following HIE. Preclinical studies demonstrate sex-specific differences in inflammatory responses and neuronal vulnerability, indicating that males and females may respond differently to both hypoxic injury and neuroprotective interventions. Consequently, future therapeutic strategies should incorporate sex as a critical biological variable to optimize treatment efficacy. Additionally, neonatal responses to neuroprotective agents cannot be directly extrapolated from adult models, as developmental differences in brain maturation, receptor expression, and hepatic and renal drug metabolism substantially affect treatment response and clearance. Recognizing these distinctions is essential for refining targeted therapies and improving long-term outcomes in neonates with HIE [28-31].

Collectively, findings from these trials and subsequent meta-analyses demonstrate that therapeutic hypothermia confers durable survival benefits and reduces the risk of death and severe neurodevelopmental impairment, thereby substantially improving long-term prognosis in infants with moderate to severe HIE.

Discussion

Based on the pathophysiological mechanisms and clinical stages of HIE described in this review, it is evident that HIE represents a progressive and multifaceted disease process rather than a single static injury. The initial hypoxic-ischemic insult triggers a cascade of cellular and molecular events, including energy failure, excitotoxicity, oxidative stress, inflammation, and apoptosis, which evolve across distinct phases and ultimately shape clinical presentation and long-term outcomes. The severity of these processes is reflected in the clinical spectrum of mild, moderate, and severe encephalopathy, emphasizing the close relationship between underlying pathophysiology and neurological staging.

Understanding this relationship has direct implications for diagnosis and management. Early clinical staging provides an initial framework for severity assessment; however, the overlap between stages and the presence of abnormal neurophysiological and imaging findings even in mild cases highlight the limitations of clinical examination alone. Integration of EEG monitoring and advanced neuroimaging allows for more accurate characterization of injury extent and timing, supporting earlier risk stratification and more informed therapeutic decision-making [11,12].

Therapeutic hypothermia targets several key components of the secondary injury phase by reducing metabolic demand, attenuating excitotoxicity, and limiting inflammatory responses. Evidence from randomized clinical trials demonstrates that this intervention improves survival and reduces severe neurodevelopmental disability in infants with moderate to severe HIE. Nevertheless, persistent neurological impairment among survivors indicates that hypothermia does not fully address all mechanisms of injury, particularly those associated with delayed neuronal loss and impaired neural repair [2,3,17].

Supportive care remains essential throughout all stages of HIE, as maintenance of physiological stability directly influences the progression of secondary injury. Careful regulation of oxygenation, hemodynamics, glucose levels, temperature, and nutrition plays a critical role in mitigating further neuronal damage and supporting recovery. Long-term outcomes are further influenced by early access to multidisciplinary follow-up and rehabilitative services, which address emerging motor, cognitive, and sensory deficits [18,19].

Future directions in HIE management must therefore build upon an integrated understanding of pathophysiology and clinical staging. Emerging neuroprotective and regenerative therapies aim to extend the therapeutic window beyond the acute phase and to promote endogenous repair mechanisms that are not fully addressed by hypothermia alone. Advances in biomarker development, individualized risk assessment, and combination treatment strategies may enable more precise targeting of therapies according to disease stage and injury profile. Continued translational research and long-term outcome studies are essential to refine prevention strategies and reduce the lifelong neurological burden associated with HIE [26-31].

Prevention and future directions of HIE

Prevention of HIE focuses on early identification of perinatal risk factors, optimization of obstetric and intrapartum care, and prompt postnatal interventions aimed at limiting secondary neuronal injury. Although therapeutic hypothermia remains the established standard of care, its effectiveness is constrained by a narrow therapeutic window that requires initiation within the first six hours of life. This limitation underscores the need for adjunctive strategies capable of extending neuroprotection beyond the acute phase of injury. Growing evidence indicates that cell-based therapies, particularly the administration of autologous umbilical cord blood stem cells, may provide an expanded therapeutic window by reducing apoptosis, oxidative stress, and excitotoxic injury while simultaneously promoting endogenous repair mechanisms. Experimental models and early clinical studies suggest that these approaches may enhance neuronal survival and facilitate regenerative processes that are not fully addressed by hypothermia alone. Consequently, future directions in the management of HIE are increasingly focused on combination therapies that integrate therapeutic hypothermia with regenerative and neuroprotective interventions. This shift is accompanied by efforts to develop standardized treatment protocols, reliable biomarkers for patient selection, and structured long-term outcome monitoring. Ongoing translational research and well-designed clinical trials are essential to determine the safety, efficacy, and optimal timing of these emerging therapies, with the overarching goal of reducing long-term neurological disability in affected infants [32].

## Conclusions

HIE remains a major cause of neonatal mortality and long-term neurological morbidity despite advances in perinatal care. Outcomes depend on injury mechanisms, clinical severity, and the timing of diagnosis and intervention. Therapeutic hypothermia improves survival but does not eliminate long-term neurological impairment. Comprehensive supportive care and structured follow-up, therefore, remain essential. Future progress will rely on earlier risk identification, improved biomarkers, and adjunctive neuroprotective strategies alongside hypothermia protocols. Continued multidisciplinary collaboration and long-term monitoring are necessary to reduce the lifelong burden of HIE and improve the quality of life for affected infants and their families.

## References

[1] Yang M, Wang K, Liu B, Shen Y, Liu G (2025). Correction to: hypoxic-ischemic encephalopathy: pathogenesis and promising therapies. Mol Neurobiol.

[2] Pappas A, Milano G, Chalak LF (2023). Hypoxic-ischemic encephalopathy: changing outcomes across the spectrum. Clin Perinatol.

[3] Selway LD (2010). State of the science: hypoxic ischemic encephalopathy and hypothermic intervention for neonates. Adv Neonatal Care.

[4] Greco P, Nencini G, Piva I (2020). Pathophysiology of hypoxic-ischemic encephalopathy: a review of the past and a view on the future. Acta Neurol Belg.

[5] Pisani F, Orsini M, Braibanti S, Copioli C, Sisti L, Turco EC (2009). Development of epilepsy in newborns with moderate hypoxic-ischemic encephalopathy and neonatal seizures. Brain Dev.

[6] Papazian O (2018). Neonatal hypoxic-ischemic encephalopathy (Article in Spanish). Medicina (B Aires).

[7] Mota-Rojas D, Villanueva-García D, Solimano A, Muns R, Ibarra-Ríos D, Mota-Reyes A (2022). Pathophysiology of perinatal asphyxia in humans and animal models. Biomedicines.

[8] Verklan MT (2009). The chilling details: hypoxic-ischemic encephalopathy. J Perinat Neonatal Nurs.

[9] Ranjan AK, Gulati A (2023). Advances in therapies to treat neonatal hypoxic-ischemic encephalopathy. J Clin Med.

[10] Adstamongkonkul D, Hess DC (2017). Ischemic conditioning and neonatal hypoxic ischemic encephalopathy: a literature review. Cond Med.

[11] Arnautovic T, Sinha S, Laptook AR (2024). Neonatal hypoxic-ischemic encephalopathy and hypothermia treatment. Obstet Gynecol.

[12] Sarnat HB, Sarnat MS (1976). Neonatal encephalopathy following fetal distress. A clinical and electroencephalographic study. Arch Neurol.

[13] Khot S, Tirschwell DL (2006). Long-term neurological complications after hypoxic-ischemic encephalopathy. Semin Neurol.

[14] Shaligram R, Garud BP, Malwade S (2024). Risk factors and predictors of outcomes in hypoxic-ischemic encephalopathy in neonates. Cureus.

[15] Caramelo I, Coelho M, Rosado M (2023). Biomarkers of hypoxic-ischemic encephalopathy: a systematic review. World J Pediatr.

[16] Sahin O, Colak D, Yavanoglu Atay F, Guran O, Mungan Akin I (2025). Evaluation of prognostic findings in newborns with hypoxic ischemic encephalopathy: 5-year experience. Ther Hypothermia Temp Manag.

[17] Mietzsch U, Radhakrishnan R, Boyle FA, Juul S, Wood TR (2020). Active cooling temperature required to achieve therapeutic hypothermia correlates with short-term outcome in neonatal hypoxic-ischaemic encephalopathy. J Physiol.

[18] Gotchac J, De Witte E, Belaroussi Y, Guichoux J, Dienst T, Brissaud O (2025). Respiratory management in neonates with hypoxic-ischemic encephalopathy during therapeutic hypothermia: a binational analysis of current practice. Eur J Pediatr.

[19] Song D, Narasimhan SR, Huang A, Jegatheesan P (2023). Increased newborn NICU admission for evaluation of hypoxic-ischemic encephalopathy during COVID-19 pandemic in a public hospital. Front Pediatr.

[20] Molina Stornelli I, Bliznyuk N, Roig JC (2025). Nutrition in infants with hypoxic-ischemic encephalopathy: insights from a single-center experience on parenteral and enteral feeding during therapeutic hypothermia. J Matern Fetal Neonatal Med.

[21] Martinovski H, Khanal L, Kraft D, Natarajan G (2025). Enteral feeding in neonatal hypoxic-ischemic encephalopathy. Am J Perinatol.

[22] Gale C, Longford NT, Jeyakumaran D (2021). Feeding during neonatal therapeutic hypothermia, assessed using routinely collected National Neonatal Research Database data: a retrospective, UK population-based cohort study. Lancet Child Adolesc Health.

[23] Rüegger CM, Davis PG, Cheong JL (2017). Xenon as an adjuvant to therapeutic hypothermia in near term and term newborns with hypoxic ischaemic encephalopathy. Cochrane Database Syst Rev.

[24] Suh ES (2021). Recent studies are focus on the new treatments for hypoxicischemic encephalopathy (HIE) and long-term outcomes in later childhood and adolescence in children with a history on HIE. Clin Exp Pediatr.

[25] Davidson JO, Wassink G, van den Heuij LG, Bennet L, Gunn AJ (2015). Therapeutic hypothermia for neonatal hypoxic-ischemic encephalopathy: where to from here?. Front Neurol.

[26] Huntingford SL, Boyd SM, McIntyre SJ, Goldsmith SC, Hunt RW, Badawi N (2024). Long-term outcomes following hypoxic ischemic encephalopathy. Clin Perinatol.

[27] Cainelli E, Vedovelli L, Mastretta E, Gregori D, Suppiej A, Bisiacchi PS (2021). Long-term outcomes after neonatal hypoxic-ischemic encephalopathy in the era of therapeutic hypothermia: a longitudinal, prospective, multicenter case-control study in children without overt brain damage. Children (Basel).

[28] Korf JM, McCullough LD, Caretti V (2023). A narrative review on treatment strategies for neonatal hypoxic ischemic encephalopathy. Transl Pediatr.

[29] Mirza MA, Ritzel R, Xu Y, McCullough LD, Liu F (2015). Sexually dimorphic outcomes and inflammatory responses in hypoxic-ischemic encephalopathy. J Neuroinflammation.

[30] Smith AL, Alexander M, Rosenkrantz TS, Sadek ML, Fitch RH (2014). Sex differences in behavioral outcome following neonatal hypoxia ischemia: insights from a clinical meta-analysis and a rodent model of induced hypoxic ischemic brain injury. Exp Neurol.

[31] Al Mamun A, Yu H, Romana S, Liu F (2018). Inflammatory responses are sex specific in chronic hypoxic-ischemic encephalopathy. Cell Transplant.

[32] Nabetani M, Shintaku H, Hamazaki T (2018). Future perspectives of cell therapy for neonatal hypoxic-ischemic encephalopathy. Pediatr Res.

